# Comparative Molecular Dynamics Simulation of Hepatitis C Virus NS3/4A Protease (Genotypes 1b, 3a and 4a) Predicts Conformational Instability of the Catalytic Triad in Drug Resistant Strains

**DOI:** 10.1371/journal.pone.0104425

**Published:** 2014-08-11

**Authors:** Mitchell Kramer, Daniel Halleran, Moazur Rahman, Mazhar Iqbal, Muhammad Ikram Anwar, Salwa Sabet, Edward Ackad, Mohammad Yousef

**Affiliations:** 1 Department of Physics, College of Arts and Sciences, Southern Illinois University Edwardsville, Edwardsville, Illinois, United States of America; 2 Drug Discovery and Structural Biology group, Health Biotechnology Division, National Institute for Biotechnology and Genetic Engineering (NIBGE), Faisalabad, Pakistan; 3 Department of Zoology, Faculty of Science, Cairo University, Giza, Egypt; 4 Biophysics Department, Faculty of Science, Cairo University, Giza, Egypt; Centro de Biología Molecular Severo Ochoa (CSIC-UAM), Spain

## Abstract

The protease domain of the Hepatitis C Virus (HCV) nonstructural protein 3 (NS3) has been targeted for inhibition by several direct-acting antiviral drugs. This approach has had marked success to treat infections caused by HCV genotype 1 predominant in the USA, Europe, and Japan. However, genotypes 3 and 4, dominant in developing countries, are resistant to a number of these drugs and little progress has been made towards understanding the structural basis of their drug resistivity. We have previously developed a 4D computational methodology, based on 3D structure modeling and molecular dynamics simulation, to analyze the active sites of the NS3 proteases of HCV-1b and 4a in relation to their catalytic activity and drug susceptibility. Here, we improved the methodology, extended the analysis to include genotype 3a (predominant in South Asia including Pakistan), and compared the results of the three genotypes (1b, 3a and 4a). The 4D analyses of the interactions between the catalytic triad residues (His57, Asp81, and Ser139) indicate conformational instability of the catalytic site in HCV-3a and 4a compared to that of HCV-1b NS3 protease. The divergence is gradual and genotype-dependent, with HCV-1b being the most stable, HCV-4a being the most unstable and HCV-3a representing an intermediate state. These results suggest that the structural dynamics behavior, more than the rigid structure, could be related to the altered catalytic activity and drug susceptibility seen in NS3 proteases of HCV-3a and 4a.

## Introduction

HCV is a worldwide health concern with severe consequences. Globally, HCV is estimated to affect around 3% of the world's population, counting to approximately 170 million people [1]. While it may remain asymptomatic for years, it can lead to serious liver diseases, which include cirrhosis or hepatocellular carcinoma [2]. As with all viruses, HCV is prone to genetic mutations that lead to multiple reproducible variants. Seven genotypes of HCV with various subtypes have been discovered around the world [3].

The genotype HCV-1 is common in America, Europe, and Japan. The subtype HCV-1a is predominant in North American and Northern Europe whereas HCV-1b is the most common subtype in Japan and Eastern Europe [4]. Additional countries where HCV infection rates are very high are Egypt (15% of population, 18 million people) and Pakistan (4.8%, 8.5 million) [5], [6]. Approximately 90% of those infected in Egypt carry the genotype 4, with subtype 4a (HCV-4a) predominating [7]–[9]. In Pakistan, around 67% of the HCV infections are due to genotype 3, with subtype 3a (HCV-3a) being the most common [10].

Genotype 1 has been the focus of intensive investigations over decades and a variety of effective antiviral drugs and/or inhibitors have been developed [11]–[13]. Conversely, variants that are predominant in developing countries have not received much attention [14].

As a result of the crucial role of the nonstructural protein 3 (NS3) in the replication cycle of HCV, the protease domain of NS3 has been an attractive target for direct-acting antiviral agents [15]. The NS3 protease cleaves four downstream sites in the HCV polyprotein and is characterized as a serine protease with a chymotrypsin-like fold, which is activated by the NS4A cofactor [16]. Similar to chymotrypsin, the catalytic triad of the HCV NS3 protease is made of three essential residues, histidine-57, aspartic acid-81, and serine-139 [17]. These three residues are collectively known as the catalytic triad and will perform general acid-base catalysis on target peptides. In summary, a charge relay system is formed in which the carboxylic group of D81 forms a hydrogen bond with N*δ*1 of H57 [18]. This event increases the p*K*a of the histidine side chain from 7 to about 12 [17], [19]. Consequently, H57 deprotonates the hydroxyl group of the S139 side chain and a proton shuttles to Nε2 of H57 [18]. The Oγ of S139 then nucleophilically attacks the carbonyl carbon of a substrate's scissile bond resulting in the formation of an oxyanion-containing tetrahedral intermediate [18], [20]–[22]. At this point, the protonated H57 acts as a general acid assisting in the collapse of the tetrahedral intermediate and the cleavage of the substrate [18], [22]. Zinc, which is a part of NS3 protease, plays an important role in the structural stability of the protease by enthalpically disfavoring protein denaturation [23]. Additionally, a bound peptide cofactor (NS4A) increases the protease activity by nearly 1000-fold [24]. It is noteworthy to mention that HCV-3a and HCV-4a NS3 proteases exhibit a several fold decrease in catalytic efficiency relative to that of HCV-1b [2]. This implies a possible correlation between the catalytic efficiency of the NS3 protease and its responsiveness to inhibition, at least by the linear inhibitor Telaprevir [25], [26]. The differential susceptibilities of different drugs to protease variants in relation to their enzymatic activities have been investigated [27]–[32].

The commercially available linear NS3 protease inhibitors Telaprevir and Boceprevir are shown to be effective against HCV-1 [33], [34]. Furthermore, HCV-3a and HCV-4a NS3 proteases show several fold resistivity to inhibition to Telaprevir compared to HCV-1b, with HCV-4a being the most resistant [25]. Additionally, the newly developed macrocyclic NS3 protease inhibitor Danoprevir has been shown to be effective against genotypes 1b and 4a, but not as effective against genotype 3a [25], [35]. Another macrocylic drug, Simeprevir, has been shown to be somewhat effective in all HCV genotypes, but is most effective against HCV 1a and b [35], [36]. Very recently, the drug Sofosbuvir, which inhibits a different HCV target protein (NS5B polymerase), has been approved by the FDA to treat genotypes 1 through 4 [37]. Although Simeprevir and Sofosbuvir provide a way to treat multiple genotypes of HCV, the cost of the typical 3 month treatment ($66,000 and $84,000 respectively), is too expensive for use in developing countries [38], [39]. In addition, due to the high mutability of the virus, a sub-type can emerge during the course of treatment, resisting the administered antiviral drug [40] and complicating the treatment regimen.

In our earlier work [26] investigating the drug resistivity in HCV-4a, we developed a computational methodology to analyze, in 4D, the active site geometry in HCV NS3 protease. The results showed that both proteases share very similar rigid and overall dynamics features. Conversely, both exhibit significantly different local dynamics and distance distribution profiles, in peak values and broadness, at the catalytic triad.

Here we have improved the methodology further and extended our investigation to include the drug resistant genotype HCV-3a. Our data, consistent with our previous report, suggest that genotype-dependent structural dynamics could play a significant role in the stability of the catalytic triad, and possibly, in drug response among HCV genotypes. The results show that the divergent dynamics behavior of the catalytic triad in the NS3 protease of genotype 3a, represent an intermediate state between that of genotype 1b (most stable) and genotype 4a (most unstable). This correlates well with their reported catalytic activities and drug susceptibilities to the linear inhibitor Telaprevir [25]. Therefore, our comparative investigation reported here, illuminates a possible variant-dependent pathway from an active/drug responsive protease to a weakly active/drug resistive one, an understanding with implications in catalysis and drug design.

## Results and Discussion

**Figure 1:**
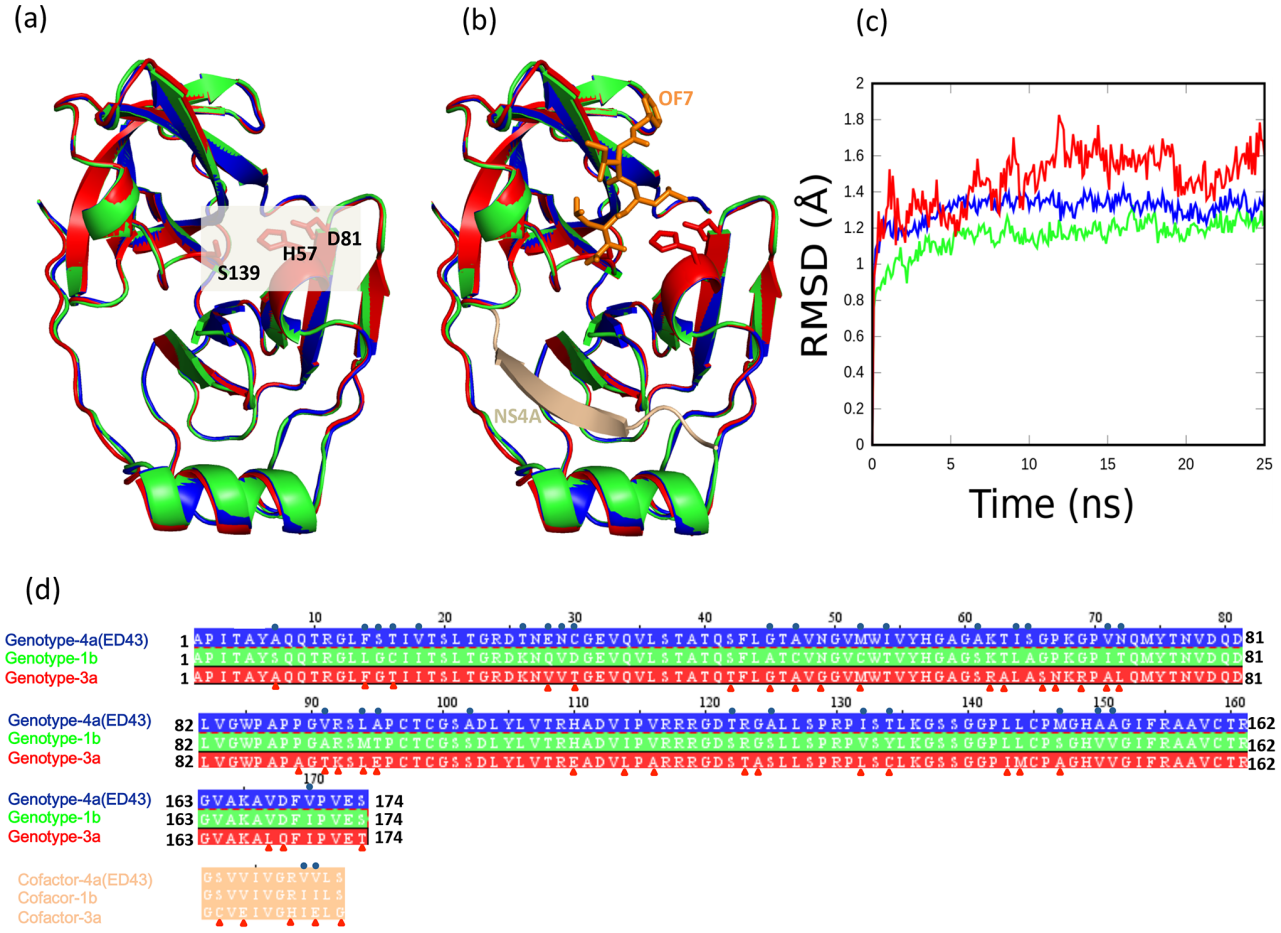
Comparison between the threading models of HCV-4a, HCV-3a and the crystal structure of HCV-1b NS3 protease. (a) Both model structures of HCV-4a (blue) and HCV-3a (red) are superimposed onto the template structure of HCV-1b (PDB: 1dy8), shown in green. The gray box highlights the catalytic triad (H57, D81, and S139). (b) Same as (a) with the addition of the cofactor (NS4A), in beige, and an example inhibitor shown for orientation purposes (OF7, 1dy8), in orange. (c) Residue-average RMSD of Cα atoms for the model of HCV-4a (blue), the model of HCV-3a (red) and HCV-1b proteases crystal structure (green) during the course of simulation. (d) The amino acid sequences of HCV-4a, HCV-3a, and HCV-1b NS3 proteases, as well as the corresponding cofactors shown with the same colors used in (a) and (b). Sequence variability between HCV- 4a and 1b are indicated by blue circles and variability between HCV-3a and 1b are indicated by red triangles.

HCV-3a and HCV-4a NS3 proteases share 80% and 83% sequence identities respectively, with HCV-1b protease (figure 1(d)). The HCV-3a and HCV-4a protease structure models superpose very well on the threading template structure (HCV-1b, PDB-ID:1dy8), along with nearly identical structural features (figures 1(a,b)). When the three structures superpose, the RMSD in back-bone positions is about 0.3 Å and none of the 174-threaded amino acids fall within the disallowed Ramachandran area and no steric clashes or stereochemical outliers were detected (see Methods).

The three catalytic residues H57, D81, and S139 are located in a crevice between the two protease β-barrels as shown in (figure 1(a)) [41]–[43]. The active site is nonpolar and shallow [18]. The central region of NS4A is buried almost completely inside the NS3 protease and serves as a cofactor for proper folding of the protease (figure 1(b)) [41]. The rigid structures indicate that access to the active site is nearly identical in the structural models (HCV-3a and HCV-4a) and template (HCV-1b) (figures 1(a,b)). Molecular dynamics simulations predict that both HCV-4a and HCV-1b proteases share more or less similar average RMSD in the Cα positions, around ∼1.2 Å at equilibrium (figure 1(c)). However, the average RMSD in the Cα positions for HCV-3a protease is higher (∼1.6 Å). This implies that the main chain of HCV-3a protease, as a whole, experiences more fluctuations compared to that of the two other genotypes (1b and 4a). Interestingly, as will be shown later, this malleability in HCV-3a protease, which is even greater than the barely functioning genotype 4a, does not propagate fully to the catalytic triad region. In this regard, HCV-3a represents an interesting case where the conformational stability of the catalytic region of the enzyme is somehow “shielded” against an overall positional instability of the protein.

**Figure 2:**
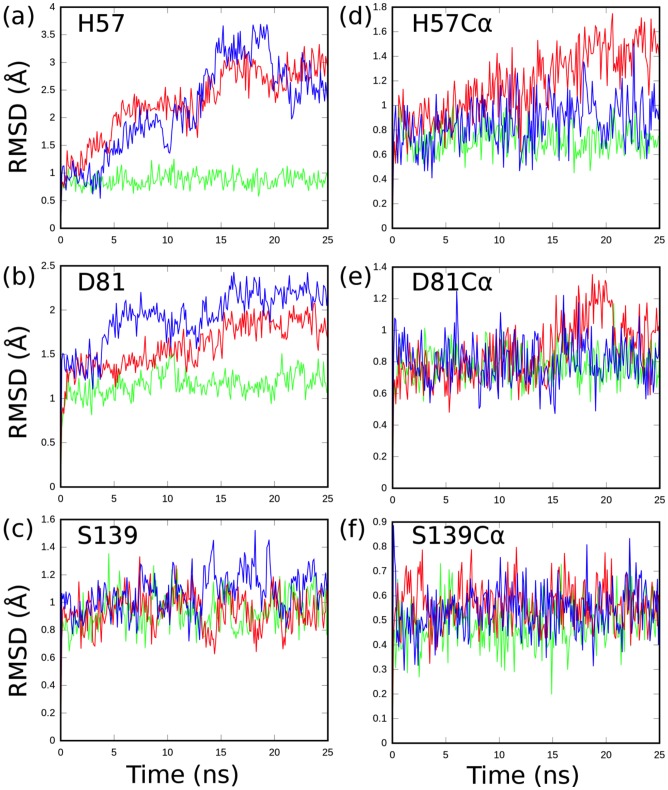
Comparison of the dynamics behavior of the catalytic triad residues between the threading models HCV-4a (blue) and HCV-3a (red) and the crystal structure of HCV-1b (green) proteases. RMSD values for each catalytic residue are shown for the entire residue (a, b, c) and the corresponding alpha carbons (d, e, f).

**Figure 3:**
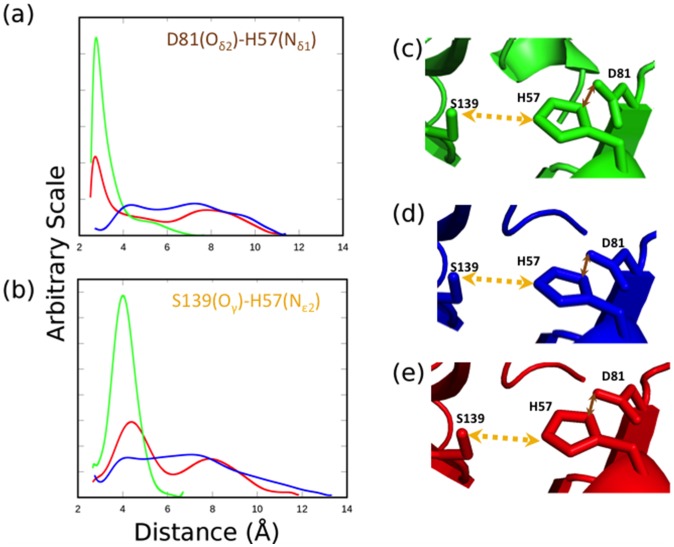
Dynamics behavior within the catalytic triad site of the threading models (HCV-4a, blue and HCV-3a, red) and the template (HCV-1b, green) proteases. The distance distribution profiles between Oδ2 of residues D81 and Nδ1 of H57 (a) and between Oγ of residue S139 and Nε2 of residue H57 (b), during the stimulation. Orange and brown arrows indicate the selected distances in the rigid structures of both the models (HCV-4a, d and HCV-3a, e) and the template (HCV-1b, c)

**Figure 4:**
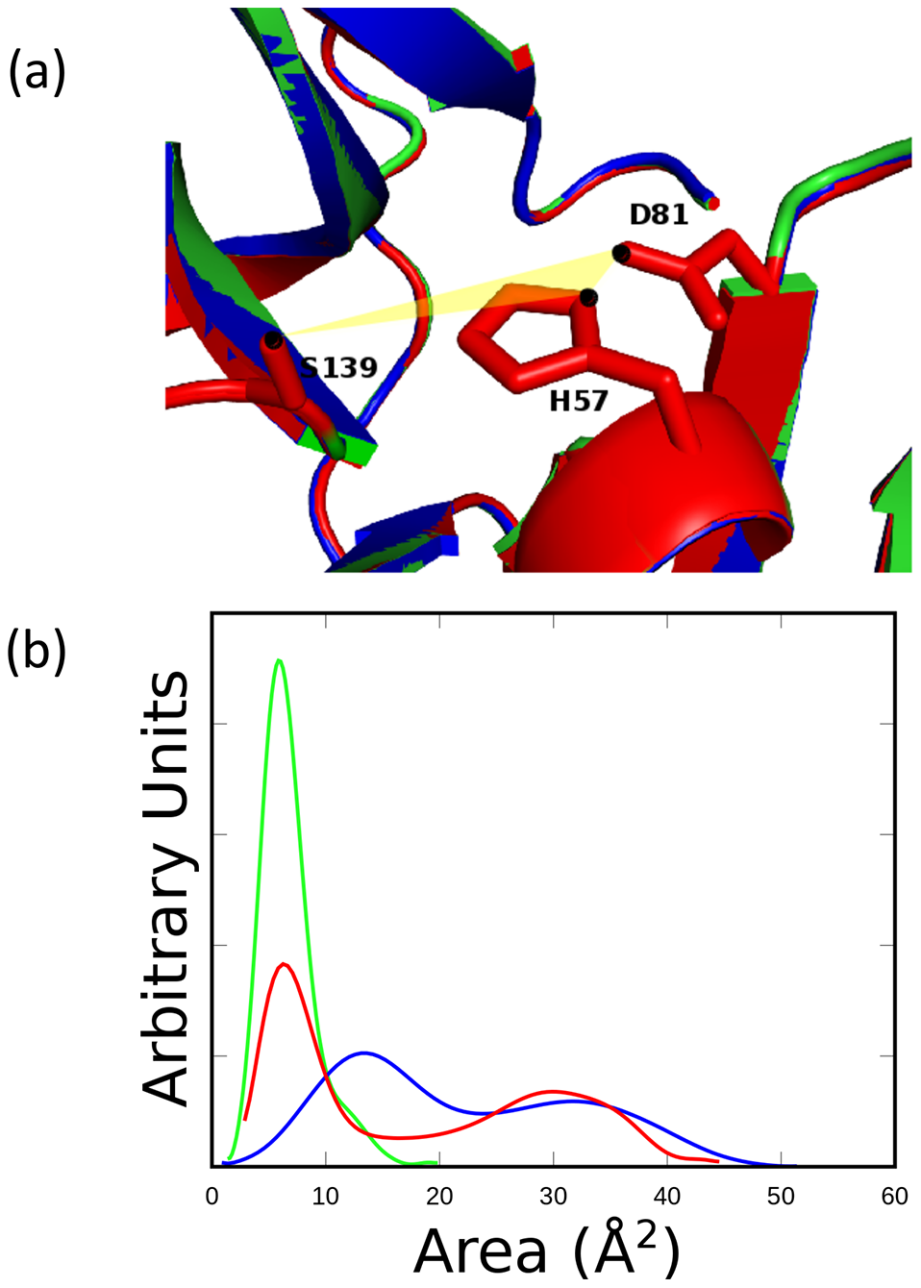
The collective dynamic behavior of the catalytic region expressed in terms of the area of a triangle (yellow) with vertices on each catalytic residue as indicated by black dots in (a). (b) The distribution profile of the area of the triangle connecting the catalytic residues in the models (HCV-4a, blue and HCV-3a, red) and the template (HCV-1b, green) structures.

Locally, molecular dynamics simulations revealed a strain-dependent, gradually divergent dynamics behavior within the catalytic triad region, with HCV-1b being the most stable, the HCV-4a the most divergent and HCV-3a representing an intermediate state, (figures 2, 3 and 4). These dynamic differences seem to correlate well with the differences in catalytic activities and drug susceptibilities to Telaprevir seen in the three genotypes [2], [25]. This result strongly suggests that the local dynamics within the triad region in the NS3 protease could be used as a direct predictive measure for HCV pan-genotype drug susceptibilities.

### 4D simulation of the interactions between the catalytic residues

Following the same methodology we reported on previously [26], we have investigated the local positional dynamics of the catalytic triad residues during the course of simulation and used the distance distribution profiles of catalytically relevant distances as indicators of the 4D variations.

The alpha carbons (Cα) of the catalytic residues D81 and S139 exhibit somewhat similar dynamics throughout the simulations for the three genotypes (1b, 3a and 4a). However, the Cα of the catalytic residue H57 of the HCV-4a and HCV-3a shows a slight to moderate increase in RMSD of ∼0.2 Å and 0.7 Å respectively, relative to that of HCV-1b (figures 2(d–f)).

As entire residues, H57 and D81 in both HCV-4a and HCV-3a models, demonstrate dynamics behavior divergent from that of the template HCV-1b (figures 2(a,b)). The RMSD of H57 in the HCV-3a model varies from that predicted in the HCV-1b template by up to 1.5 Å at certain points during the simulation (figure 2(a)). A very similar trend is observed for HCV-4a. A slight increase in the Cα dynamics of H57 in HCV-4a is associated with a significant increase in the dynamics of the entire residue (figures 2(a,d)). Therefore, one should expect a much higher RMSD for H57 (as an entire residue) in HCV-3a, if only the corresponding Cα instability is considered (figure 2(d)). The observation that H57 (as an entire residue) shows similar RMSD pattern in both HCV-3a and HCV-4a (in spite of the relative stability of the Cα in HCV-4a) points to additional stabilizing interactions acting on H57 in HCV-3a. This is evident in HCV-3a from the intermediate RMSD dynamics of D81 residue (figure 2(b)), which is known to fulfill a stabilizing role for H57 [18]. The RMSD of D81 in the HCV-3a and HCV-4a models gradually diverge from that of HCV-1b template by nearly 0.5 Å and 1 Å respectively (figure 2(b)). S139 exhibits similar dynamics behavior in the three genotypes (figures 2(c,f)), which is expected for a relatively small side chain whose movement is sterically encumbered by the nearby residues (figures 2(c,f)).

The distance distribution profiles between Nε2 of H57 and Oγ of S139, as well as between Nδ1 of H57 and Oδ2 of D81, of the template (HCV-1b) and models (HCV-3a and HCV-4a) vary widely in both peak value and breadth. In the template structure (HCV-1b), the distance between Oδ2 of D81 and Nδ1 of H57 (figure 3(c)) exhibits a sharp distribution with a peak value around 3 Å (figure 1(a)). In the model HCV-4a (figure 3(d)), the corresponding distance distribution is bimodal, much broader, and distributed around 4.5 and 7.5 Å (figure 3(a)). It is noteworthy to mention that in our previous report [26], the 4.5 Å peak was not conspicuous. With the improved methodology in the current analysis (see Methods), this peak becomes clearer. In the model HCV-3a (figure 3(e)) a broader bimodality (∼70% of the distribution) still dominates, with the recovery of a sharp peak around 3 Å, overlapping with that of HCV-1b peak. It is interesting that the distribution for the HCV-3a model is almost a mixture of that of HCV-1b and HCV-4a. Furthermore, the distance distribution between Oγ of S139 and Nε2 of H57 in the template HCV-1b shows a peak value at around 4 Å (figures 3(b,c)), while the corresponding distribution in the model HCV-4a is broader and bimodal (figures 3(b,d)). Again, the distribution in HCV-3a is a mixture of HCV-1b and HCV-4a shifted by about 0.5 Å (figures 3(b,e)).

The collective dynamic behavior of the three catalytic residues as vertices of a triangle is analyzed (figures 4(a,b)). This is to gain an insight into the respective relative positions of the three residues simultaneously. The atoms chosen were Oδ2 of D81, Nδ1 of H57 and Oγ of S139. The choice of Nδ1 over Nε2 of H57 was to include conformations in which the H57 has rotated in such a way that Oδ2 of D81 and Nδ1 of H57 can no longer hydrogen bond. While more than three atoms are involved in the hydrogen bonding network, the vertices of the triangle collectively probe the hydrogen bonding distances. Thus, any other choice of vertices would only shift the values, not the distribution. The distribution profiles of the area of the triangle during the course of simulation indicate a uni-modal sharp peak in HCV-1b, a broad bimodal distribution in HCV-4a and a hybrid behavior in HCV-3a. The area of the triangle somehow represents a “catalytic plane” whose distribution profile could be predictive of distortions of optimal catalytic geometries. In this sense, HCV-1b is predicted to be the most stable (most active) and HCV-4a is the least stable (least active) while HCV-3a represents an intermediate state. This is consistent with the observation that the catalytic activity of HCV-4a NS3 protease is several orders of magnitude less than that of HCV-1b, while the catalytic activity of HCV-3a is still less than that of HCV-1b, but not as hampered as that of HCV-4a [2].

The same trend was observed in the genotype-dependent drug susceptibility seen in HCV against the linear inhibitor Telaprevir; HCV-1b is the most responsive (least resistive), HCV-4a the least responsive (most resistive) and HCV-3a representing an intermediate state [25]. Telaprevir is a linear inhibitor that fits within the natural substrate' binding site “envelope”. Therefore, it is expected that variations (whether rigid or dynamic) altering inhibitor binding will simultaneously interfere with the binding of substrate; thus, impact the enzymatic activity [44]. However, other inhibitors protrude from the substrate binding envelope, interacting with sites remote from the substrate binding site. Variations occurring at these sites incur drug resistivity, with little effect on the catalytic activity [27], [29]-[31]. In general, drug susceptibilities to protease variants depend on both the 3D location of the variation (mutation) sites, and the stereochemical structure and conformation of the inhibitor. However, dissecting the molecular and structural basis of the differential susceptibilities of different drugs to protease variants in relation to their enzymatic activities is rather extensive, beyond the scope of this work.

Together, these data indicate that in the model HCV-3a, H57 spends less time positioned within a probable hydrogen bonding distance to both S139 and D81 compared to HCV-1b, but more time compared to HCV-4a. Thus, H57 is less likely to act as an efficient general acid–base in case of HCV-3a. The effect is more severe in case of HCV-4a where H57 is not positioned within hydrogen bonding distance with D81 and barely with S139. As mentioned before, it seems that the mode of Telaprevir binding somehow allows the protease drug responsiveness to follow the enzymatic activity trend [31].

It is important to note that the predicted divergent dynamics behavior in HCV-3a and HCV-4a (figures 3(a,b)) is completely hidden by the apparent similarity seen in the catalytic site in the rigid structures (figures 3(c–e)). Further, the global instability of the protein's backbone fails to accurately account for the stability of the triad as does the stability of the Cα of the H57. These results highlight the importance of utilizing molecular dynamics as a method of future investigations into protease activity. Furthermore, the correlation between the divergent conformational stability of the catalytic triad region with both the catalytic activity and drug resistivity seen in HCV-proteases cross genotypes, opens an interesting avenue for inquiry with potential predictive applications.

## Methods

### DNA sequencing

For HCV-4a (strain ED43), we used the amino acids sequence, as described previously [26]. To obtain nucleotide/amino acid sequence of NS3 protease from HCV 3a, RNA was extracted from blood sample of a Pakistani patient and cDNA was synthesized using first strand cDNA synthesis kit (Fermentas, cat no. K1612).

Freshly synthesized cDNA was used to amplify the full length NS3 gene using forward primer 5' TATAGGATCCATGCACCATCACCATCATCACGCCCCGATCACAGCATAC3' and reverse primer 5' GAGCAAGCTTTTAGGTGGTTACTTCCAGATCAG 3' containing the *Bam* H1 and *Hind* III sites, respectively, through a gradient PCR reaction. The amplified product was cloned in pET 11a vector and sequenced. The sequence was submitted to NCBI GenBank under the accession number JQ676838. The Research Ethics Review Committee of National Institute for Biotechnology and Genetic Engineering (NIBGE), Faisalabad, Pakistan has approved the protocols and procedures used to collect the blood samples from HCV patients. A written informed consent (as outlined in PLOS consent form) to participate in this study and publish the case details was taken from every donor.

### 3D structure prediction and validation

The 3D structure of HCV-3a and HCV-4a NS3 proteases were predicted by threading its amino acid sequence through the X-ray crystal structure of HCV-1b NS3 protease (1dy8, [45]) via the threading program LOOPP [46]. LOOPP is a fold recognition program that generates atomic coordinates of a sample molecule based on an alignment with a homologous template structure. By integrating the results from direct sequence alignment, sequence profile, threading, secondary structure, and exposed surface area prediction, the LOOPP builds main-chain and all-atom models. Nearly identical models were also obtained via homology modeling using the SWISS-MODEL Workspace [47]–[51]. The RMSD values between models obtained using LOOP and SWISS-MODEL were about 0.2 Å and 0.16 Å for HCV-3a and 4a respectively.

To build the NS4A cofactor, we superposed the model structures onto the template structure (1dy8, RMSD 0.3 Å) and built the sequence of the NS4A cofactor for the model based on the corresponding coordinates found in the template crystal structure. Similarly, a single zinc ion was manually docked at the cysteine triad C97, C99, and C145 into the model guided by the corresponding position in another structure of the template protein (1dxp) in which zinc is present [45]. With the cofactor and zinc bound, the model was energy minimized using the CCP4 program suite [52]–[53] and the GROMOS96 program, an implementation of the Swiss-pdb viewer [54].

The final models were validated using the NIH MBI Laboratory for Structural Genomics and Proteomics Structural Analysis and Verification Server. This server utilizes five programs (Procheck, What_Check, ERRAT, Verify_3D, and Prove) to analyze the stereochemical parameters and the quality of the model [55]–[59]. Additionally, CCP4 programs suite 6.0 was used for the calculation of a Ramachandran plot, structure superposition, and RMSD value calculation in addition to the evaluation of the stereochemistry [53], [60].

### Molecular dynamics simulation

The molecular dynamics simulation (MD) was performed using NAMD 2.9 under the CHARMM27 force field for proteins [61]–[63]. Initially, the 3D structures were solvated using the solvation tool in VMD [64]. The TIP3P model was used for the water molecules [65]. Lengevin dynamics for all nonhydrogen atoms with a damping coefficient of 1 ps^−1^ was used in maintaining a constant temperature of 310 K throughout the system. A constant pressure of 1 atm was maintained using a Nosé–Hoover Langevin piston with a period of 100 fs and damping timescale of 50 fs [66].

Periodic boundary conditions were used on a 61 Å cubic box with the long-range electrostatics calculated using the particle-mesh Ewald method with a grid point density of 0.92 Å^−1^. This process ensured that adjacent copies of the protease were never close enough for short-range interaction. A cut-off of 10 Å for van der Waals interactions and a switching distance of 8 Å were found to give convergent results, thus used for production runs. The solvation box was neutralized, using VMD's Autoionize plugin version 1.3, with sodium chloride placed at distances greater than 5 Å from the protease.

A time step of 1 fs was used in order to resolve the hydrogen motion of water. The initial structure was first subjected to three rounds of an 800 cycle conjugate gradient energy minimization flanked by 100 ps of MD simulation at 278 K. The system was then heated up in increments of 5 K with 100 ps of MD simulation at each temperature increment until the desired temperature of 310 K was established (the last heating increment was 2 K). This is an overly cautious stochastic heating scheme to ensure that the models explore wider space around a local minimum, given that HCV-3a and 4a proteases are predicted models, not crystal structures like HCV-1b.

The system was then simulated for 25 ns. Time frames used for the measurements within the protease were only done for frames where the protease had equilibrated: 15–25 ns. The equilibrium state of the protease was determined by the RMSD of the entire protein's backbone. For HCV-3a, longer runs (40 ns) were performed to ensure equilibration beyond 25 ns.

Multiple copies of each protease, which included the cofactor and a zinc ion (nonbonded), were run with different initial conditions to ensure that the results were well converged. All data presented are averaged over six distinct runs in order to ensure a representative sample of the parameter space the protease explores.

In order to quantify the relative positions of the three catalytic residues (H57, D81 and S139) simultaneously, three atoms were used as vertices of a triangle. The atoms chosen were Oδ2 of D81, Nδ1 of H57 and Oγ of S139. The area is calculated by 
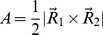
 where 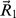 is the vector from Oγ of S139 to Nδ1 of H57 and 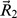 is the vector from Oγ of S139 to Oδ2 of D81. The distribution of the area of the triangle was monitored during the course of the simulation.
