# Correction: Comparative Molecular Dynamics Simulation of Hepatitis C Virus NS3/4A Protease (Genotypes 1b, 3a and 4b) Predicts Conformational Instability of the Catalytic Triad in Drug Resistant Strains

**DOI:** 10.1371/journal.pone.0111263

**Published:** 2014-10-15

**Authors:** The PLOS ONE Staff

The eighth author’s name is missing the middle initial. The correct name is Mohammad S. Yousef.
