# Correction: Comparative Molecular Dynamics Simulation of Hepatitis C Virus NS3/4A Protease (Genotypes 1b, 3a and 4a) Predicts Conformational Instability of the Catalytic Triad in Drug Resistant Strains

**DOI:** 10.1371/journal.pone.0116131

**Published:** 2014-12-19

**Authors:** The PLOS ONE Staff

## Notice of Republication

This article was republished on November 20, 2014, due to an error in the title and citation, as well as the spelling of the name of the fifth author, Muhammad Ikram Anwar. The originally published, uncorrected article and the republished, corrected article are provided here for reference.

## Supporting Information

File S1 Originally published, uncorrected article
(PDF)

File S2 Republished, corrected article
(PDF)
